# Effects of an Elevated Platform on Welfare Aspects in Male Conventional Broilers and Dual-Purpose Chickens

**DOI:** 10.3389/fvets.2021.660602

**Published:** 2021-05-31

**Authors:** Julia Malchow, Lars Schrader

**Affiliations:** Institute of Animal Welfare and Animal Husbandry, Friedrich-Loeffler-Institut, Celle, Germany

**Keywords:** animal welfare, dual-purpose strain, broiler chicken, environmental enrichment, elevated platform

## Abstract

To avoid killing day-old male chicks, one possibility is to keep dual-purpose chicken strains. Here, the hens were kept for egg production, and the roosters were kept for meat production. Both sexes had moderate performances compared to the respective hybrid chicken strains. However, until now, little has been known about whether male dual-purpose chickens may profit from enrichment in the environment in which broiler chickens are raised under conventional conditions. This study aims to further investigate the suitability of elevated structures for dual-purpose chickens (Lohmann Dual) with moderate growth and for fast-growing male broiler chickens (Ross 308). In two consecutive trials, we kept 686 Ross and 672 Dual chickens in 24 compartments (2 trials × 2 strains × 6 compartments). Half of the compartments were equipped with elevated grid platforms at a height of 50 cm (enriched group). In the other half of the compartments, no platforms were installed (control group). We analyzed the usage of the elevated platforms by scan sampling and assessed animal-based (walking ability, plumage cleanliness, and foot health) and management-based (litter quality) indicators. Both strains showed increasing use of the elevated platforms from the first week of life onwards. However, the fast-growing chickens used the elevated platform less than the slow-growing chickens. At the end of the fattening period, the birds used the elevated grids more at night than during the daytime. Slow-growing chickens kept in enriched compartments showed a better walking ability. In general, slow-growing chickens had better plumage conditions and foot health compared to fast-growing chickens. Our results show that natural behaviors such as perching can be supported by offering elevated platforms and that animal-based indicators such as walking ability can be improved, at least in slow-growing chickens. Moreover, the use of an alternative chicken strain avoids killing day-old male chicks, and in addition, these chickens show fewer animal welfare problems than a conventional fattening strain. Thus, the use of male chickens of a dual-purpose strain can substantially contribute to improving animal welfare in broiler meat production.

## Introduction

Societal discussion about the killing of day-old male layer chicks started several years ago and is still continuing. A solution that can be practically implemented has not yet been found, but there are some approaches to avoid the unnecessary killing of day-old male chicks. The sex of the embryo can be determined in the incubated egg (1), which has already been performed but has only been accessible to a small niche until now. Another alternative is to rear and later slaughter the male chickens of layer hybrids. However, this possibility is hampered by the clear antagonism between muscle growth and egg production (2), resulting in very slow growth and poor feed efficiency in male layer chickens. Dual-purpose strains can be a more efficient alternative. For example, studies have shown that certain dual-purpose strain (Lohmann Dual) hens have a moderate egg performance (250 eggs/year) in contrast to hybrid hens (320 eggs/year), but the roosters have a better growth rate (25 vs. 15 g/day) and a higher live weight at an age of 67 days (1,700 vs. 1,061 g) compared to male layer chickens (3, 4). In parallel to the moderate performance, hens of this dual-purpose strain did not show any damaging behaviors, i.e., feather pecking, resulting in complete plumage by the end of the laying period (5).

In comparison to conventional fast-growing broiler chickens, dual chickens show moderate growth rates. Rapid growth during a short fattening period is associated with certain welfare and health problems, such as impaired walking ability, reduced locomotor activity, and a high prevalence of foot pad lesions (6). In addition, conventional broiler chickens are most often kept in a barren housing environment, where a variety of species-specific behaviors can hardly be expressed. By offering elevated platforms, further behavioral patterns such as perching can be supported. Perching is performed by chickens during the daytime as well as at night. At night, perching serves as an antipredator behavior, i.e., animals seek shelter on higher places. During the daytime, preening, standing, locomotion, exploration, or resting is performed, and perching birds can escape aggressive encounters on perches (7). Furthermore, enrichment of the housing environment with possibilities for perching can positively support birds' activity (8) and walking ability (9). By using non-littered elevated platforms and increasing activity, such as locomotion, chickens have less contact with litter on the chest. By reducing the number of times chickens stay in the litter area, the moisture in the litter may be reduced due to improved ventilation. Drier litter can contribute to improved foot health (10) and possibly to cleaner plumage (11).

This study aims to further investigate the suitability of elevated platforms for both fast-growing male broiler chickens and dual-purpose chickens with moderate growth. We have already shown that broilers prefer elevated grids compared to perches (12) and frequently use elevated grids offered at a height of 50 cm (8). Thus, in a recent study, we offered an elevated grid with an area sufficient for ~60% of the chickens to both fast-growing (Ross 308) and slow-growing (Lohmann Dual) chickens. To test the effects of the platforms on certain welfare aspects, platforms were offered in only half of the test compartments.

Due to their minor activity and impaired walking ability, we hypothesized that fast-growing male broiler chickens would use the elevated platform less frequently than male dual-purpose chickens. In addition, we expected that chickens in compartments with elevated platforms would have a better walking ability due to possible training effects. Furthermore, we hypothesized better foot health in chickens from enriched compartments because usage of the grid platforms may allow drier feet when not permanently exposed to often-moist litter. However, the plumage condition of chickens in the compartments enriched with platforms will be worse because feces of chickens may drop on chickens below the elevated platform.

## Materials and Methods

### Housing Conditions

The study was conducted at the research station of the Institute of Animal Welfare and Animal Husbandry (FLI, Celle, Germany). All investigations were carried out with the approval of the Lower Saxony State Office for Consumer Protection and Food Safety (LAVES, Oldenburg, Germany, file number 33.19-42502-04-16/2108).

**Figure 1:**
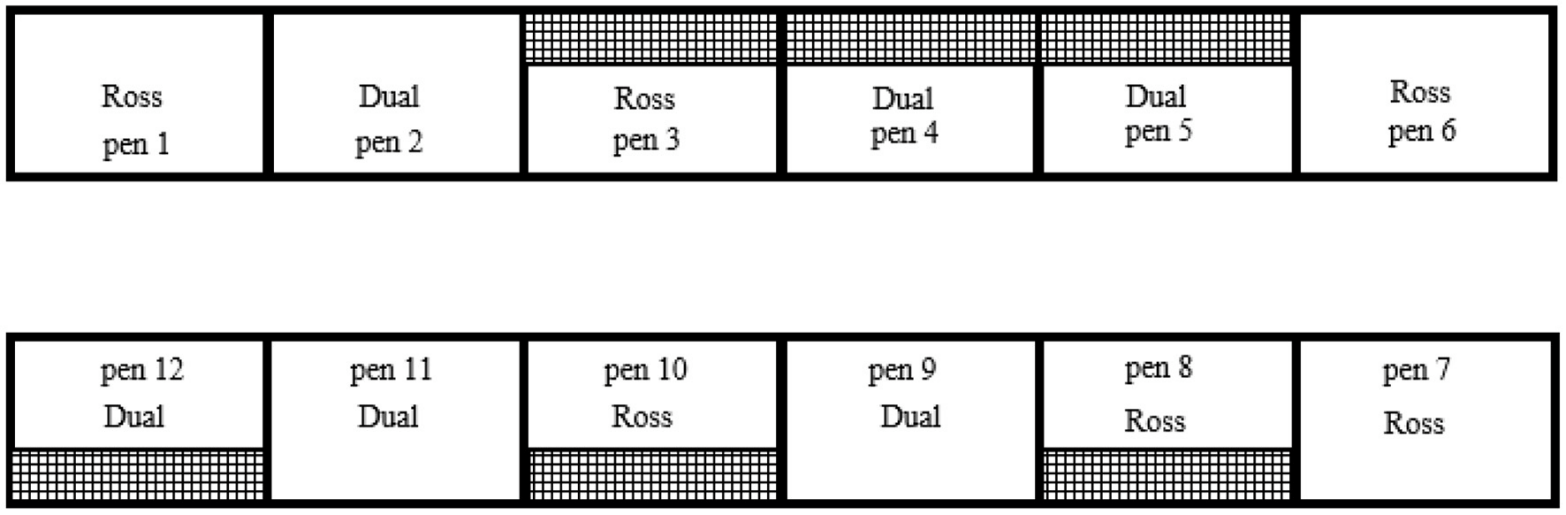
Schematic view of the allocation of strains and treatments across the 12 experimental compartment. In both trials, strains (Ross 308, Lohmann Dual) and treatments (with/without an elevated platform) were randomly assigned to the compartments, resulting in three replicates of each combination per trial. Data were collected in two trials, resulting in a total of six replicates per combination.

In two successive trials, two different broiler chicken strains, Ross 308 [Ross, fast-growing strain; Aviagen® (13)] and Lohmann Dual (Dual, slow-growing strain), were kept in groups of 56–57 birds each (depending on the total number of chickens delivered). In both trials, each group of a strain was randomly assigned to 12 experimental compartments (Figure 1), resulting in a total of 686 Ross and 672 Dual chickens. All birds were obtained as day-old male chickens from commercial hatcheries and were reared to 5 (Ross; body weight at hatch, 44.2 ± 1.3 g; live weight at slaughter, 2,051.7 ± 351.3 g) and 10 weeks of age (Dual; body weight at hatch, 41.8 ± 0.7 g live weight at slaughter, 2,237.8 ± 232.7 g). Sexes were determined in the respective hatchery by professional sorters using cloacal sexing for Dual and feather sexing for Ross (Supplementary Table 1).

The climate in the barn was controlled by an automatic ventilation and heating system according to a program set for broiler chickens. The lightening regime was artificially maintained at a minimum of 20 lx during the light period by flicker-free tube bulbs (Newlec cold white, HFT 18/840, REXEL Germany GmbH & Co. KG, Munich, Germany). During the first 3 days, the barn was lighted for 24 h, and from the fourth day of life, lighting was reduced stepwise from 3 days to 16 h/day, resulting in a dark period of 8 h. Between light and dark periods, there was a dimming phase of 15 min.

In each experimental compartment, the floor area [L (length) × W (width), 3 × 2 m] was covered with wood shavings and equipped with one feeding trough and one round water dispenser. All chickens had *ad libitum* access to water and to single-phase pelletized feed (21% crude protein, 12.90 MJ ME/kg) meeting the energy requirements for both strains.

**Figure 2:**
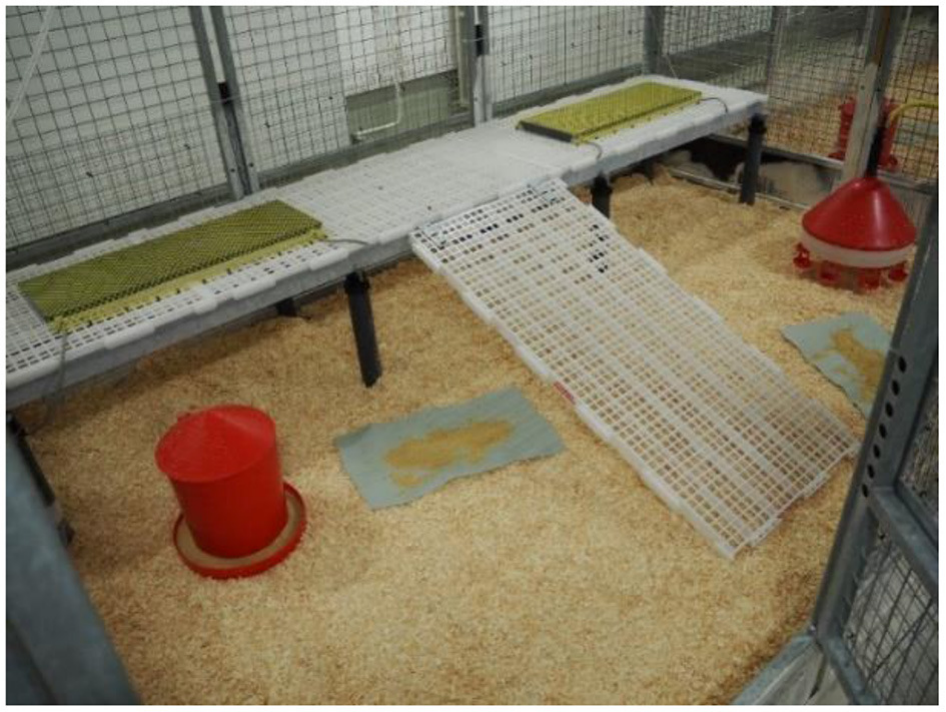
Photo of a compartment equipped with an elevated grid platform and a ramp for easy access. Platforms were installed in half of the compartments (enriched compartments). Control compartments were identical but without platforms.

In both trials, half of the compartments were equipped with elevated plastic grids (L × W, 3 × 0.6 m; mesh size, 18.7 × 20 mm; slat width, 10 mm; MIK International GmbH & Co. KG, Ransbach-Baumbach, Germany) adjacent to one of the longer partition walls of the compartments at a height of 50 cm and equipped with a ramp (L × W, 0.9 × 0.6 m; inclination angle, 29.1°; same material as the elevated grid, Figure 2). The area below the elevated platform was accessible to the birds. Compartments with elevated platforms had an additional area of 20% in relation to the floor area compared to compartments without platforms. In the following, compartments with elevated platforms are termed enriched, and the others are termed control compartments.

### Measurements and Statistical Analysis

#### Usage of Elevated Platforms

In each of the enriched compartments, an infrared video camera (Model VTC-E220IRP, color camera for corner mount with IR-LEDs; SANTEC BW AG, Ahrensburg, Germany) was installed opposite to the elevated structure to record the usage of the platforms from the first day until the end of the fattening period.

The usage of the elevated platforms was analyzed by counting the number of chickens on the grids using scan sampling in 15-min intervals every week of life throughout two successive days (Saturday and Sunday), including 64 time points during the light period. During the dark period, usage was recorded at five time points, as a former study showed less variation in the use of elevated structures during the night (12).

#### Walking Ability

We assessed the walking ability 2 and 1 day before slaughter (Ross, end of the fifth week of age; Dual, end of the tenth week of age) by two methods. Two methods were used because there was an association between them, and any differences were checked.

First, the chickens were placed in a small arena outside their home pen with two other chickens to assess the gait score after Kestin et al. (14) with a 6-point scale (0 = fluent walking without detectable abnormality for chickens, 1 = slight undefined defect in gait, 2 = definite changes and defects and waddling walk, 3 = clearly fluent gait restriction, 4 = severe gait defect, difficult to move, and 5 = unable to walk). To motivate the chicken to walk, the focus animal was placed on one side with two other chickens on the other side of the small arena. Gait scores were assessed while the chickens were walking. Directly after the assessment of gait scores, the chickens were transferred to a rotarod test (15) installed in the corridor of the barn. The test chicken was placed on a stationary rod. After the chicken grasped the rod, the rotarod test started, and the rod started to rotate after 1 s. The test was completed when the chicken left the rod actively or passively. Detailed information on the rotarod test is given in (15). As a proxy for walking ability, the latency to leave the rod in seconds was recorded.

For the chickens of both strains, we used different diameters of the rod to match the rod to the different foot sizes of the chickens (length from the middle to the back toe: Ross 308, 89.1 mm; Lohmann Dual, 105.6 mm). The diameter of the rod was 57 mm for Ross chickens and 67 mm for Dual chickens. For each strain, we tested a total of 114 chickens (114 = 19 chickens × 6 compartments). The animal sample size was calculated by power analyses (*F*-test, power = 0.8) based on previous results.

#### Weight, Plumage Cleanliness, and Foot Health

For weighing and assessment of animal-based indicators, we examined the chickens that passed the rotarod test. The weight of day-old chicks was measured at the pen level [average weight = (all chicks in one box per pen – tara value of the box)/number of chicks].

To assess plumage cleanliness and foot health, we used the Welfare Quality® (16) scoring system. The state of the plumage cleanliness of the back and chest was classified into 4 scores (0 = clear and fluffy, 1 = slightly dirty, 2 = moderately dirty, and 3 = completely dirty). The foot pads and hock burns were classified using 5 scores (0 = no evidence of foot pad dermatitis or hock burn, 1 = slight changes, 2 = moderate changes, 3 = major changes, and 4 = severe evidence of foot pad dermatitis or hock burn).

#### Litter Quality

**Figure 3:**
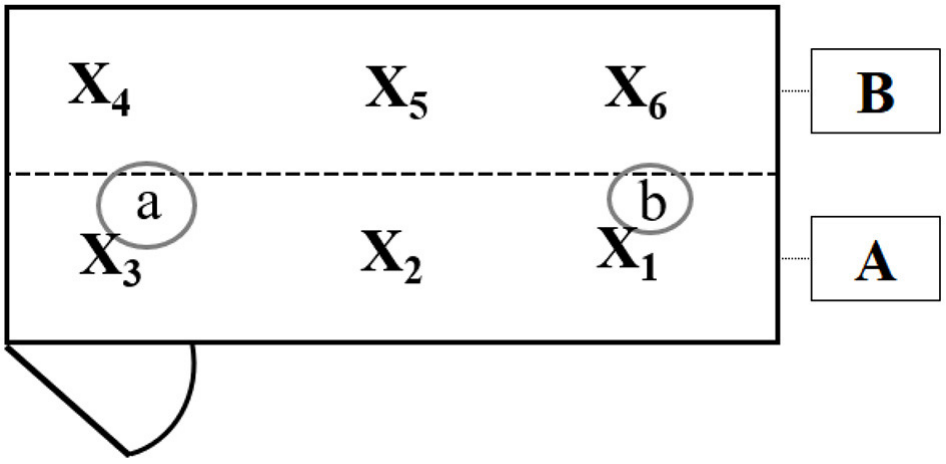
Schematic view of the areas of litter sampled and subsequently analyzed for dry matter content. Area “A” contained the feeding trough (a) and drinkers (b), and area “B” was below the elevated platform. From both areas, a mixed sample was taken, respectively (A: x_1_, x_2_, x_3_; B: x_4_, x_5_, x_6_).

After each fattening period, two mixed samples (50 g) were taken from each pen (A = three small samples from the area around the water dispenser and feed troughs, B = three small samples from the area under the elevated structure; Figure 3). To determine the relative moisture content, each sample was dried in a forced-draft oven at 105°C for 24 h [Darr method, DIN 52,183; (17)] and weighed immediately afterwards.

#### Statistical Analysis

For statistical analyses of the usage of the elevated structure, the latencies of the rotarod test, and the weight, we applied linear mixed-effect models (LMEs) using the nlme package (18) of RStudio Version 1.2.5042. To test for differences in the relative usage of elevated structures, we included the strain (Dual, Ross), weeks of age (5, 10 weeks), and time of day (dark, light periods) as fixed factors. The compartment ID nested within the trial (1, 2) was considered a random factor. Effects on latency in the rotarod test and weight were tested by LME, including the strain and treatment (enriched or control), and their interaction as fixed factors. The compartment ID nested within the trial was included as a random factor.

The relative moisture content of litter taken from the two areas (A, B) was analyzed using a linear model (LM) with treatment, strain, and their two-way interaction as fixed factors. One trial was removed from the final model, as it did not show any significant effect on the relative moisture content. Within the enriched compartments, another LM for litter quality was conducted to examine differences between the “A” and “B” positions with strain and position and their two-way interaction as fixed factors. All dependent variables were log(x + 1) transformed. In the case of significant effects of the factors, a *post-hoc* comparison with a pairwise *t*-test (Bonferroni correction) was conducted.

Effects on the gait score, plumage cleanliness score, and foot health score were tested by generalized linear mixed models (glmer) with a Poisson distribution using the package lme4 (19). To analyze differences in gait score between treatments, single models were used for each strain due to convergence issues in the model processing. The effects of treatments and strains on plumage cleanliness and foot health were tested by including the treatment and strain (except for gait score) and their interaction as fixed factors. The compartment IDs nested within the trials were considered random factors in each model. Model outputs were extracted from GLMER using the package car (20).

## Results

In the following section, we present the main results of the study (see the Supplementary Material for the whole output of the statistical models).

### Usage of Elevated Platforms

**Figure 4:**
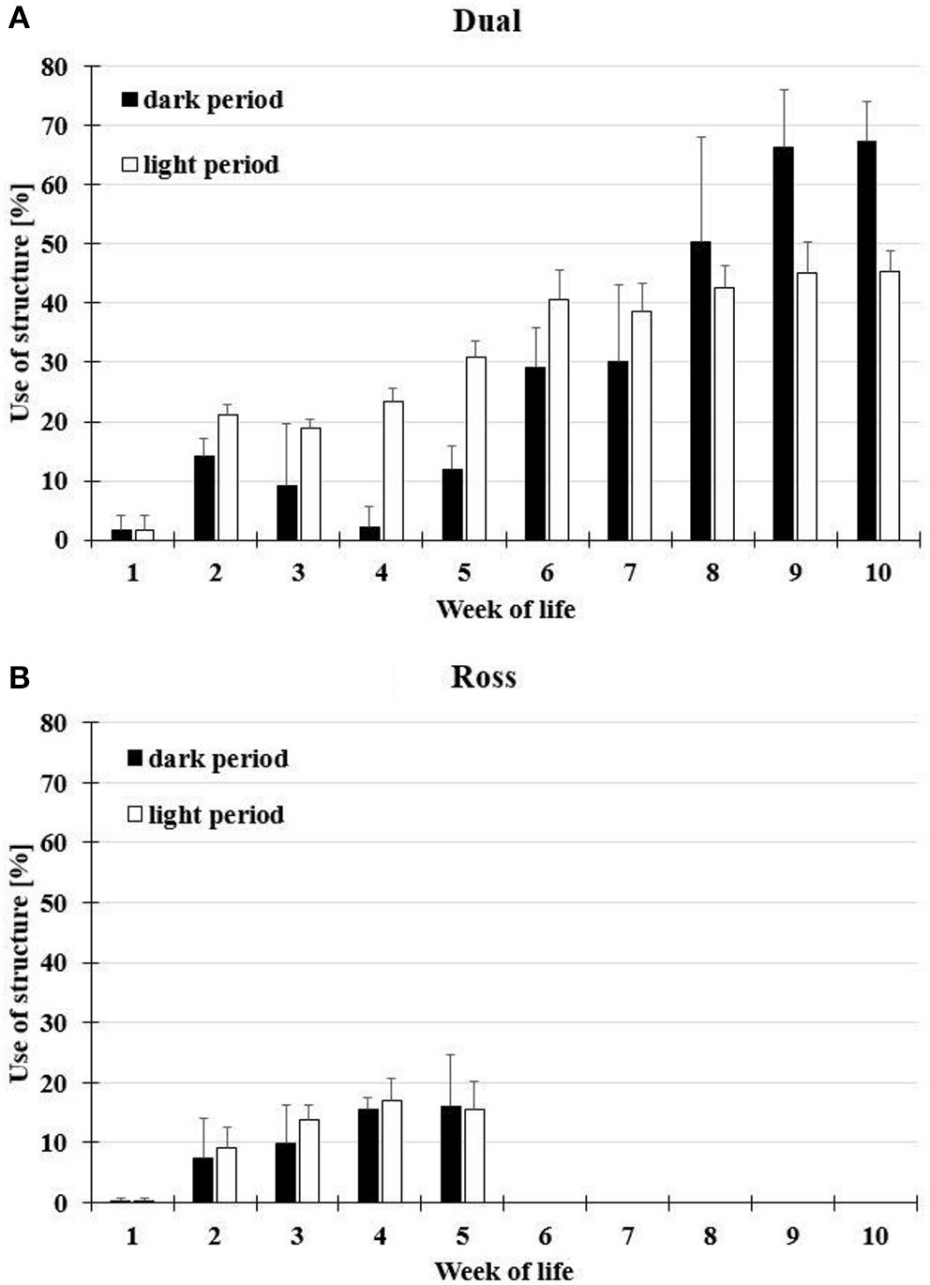
Effect of age and time of day on the usage of the elevated platforms (means ± SD) by **(A)** Dual and **(B)** Ross chickens.

The chicks can be observed on the ramp from the second day of life. On the elevated platforms, they are only from about the third day of life. The usage of elevated platforms was significantly affected by a two-fold interaction between weeks of age and time of day [*F*_(1, 163)_ = 26.12, *P* < 0.0001] and by the strain [*F*_(1, 9)_ = 199.09, *P* < 0.0001; Supplementary Table 2]. Chickens of both strains increasingly used the elevated platforms with increasing age (Figure 4). Especially in the second week of life, the platforms were used more often than in the first week of life. Dual chickens were observed more often on the elevated platforms in light compared to during the dark period until the seventh week of age. Afterwards, they used the platforms more often during the dark period. The Ross chickens showed comparable usage of the elevated platforms during the light and dark periods. However, Ross chickens used the elevated structures less than Dual chickens. The highest usage of elevated platforms was observed in Ross chickens in the fourth week of life during the light period, with a mean proportion of 17.1 ± 3.5%. Dual chickens showed the highest usage in the 10th week of life during the dark period, with a mean proportion of 67.3 ± 6.8%.

### Walking Ability

**Figure 5:**
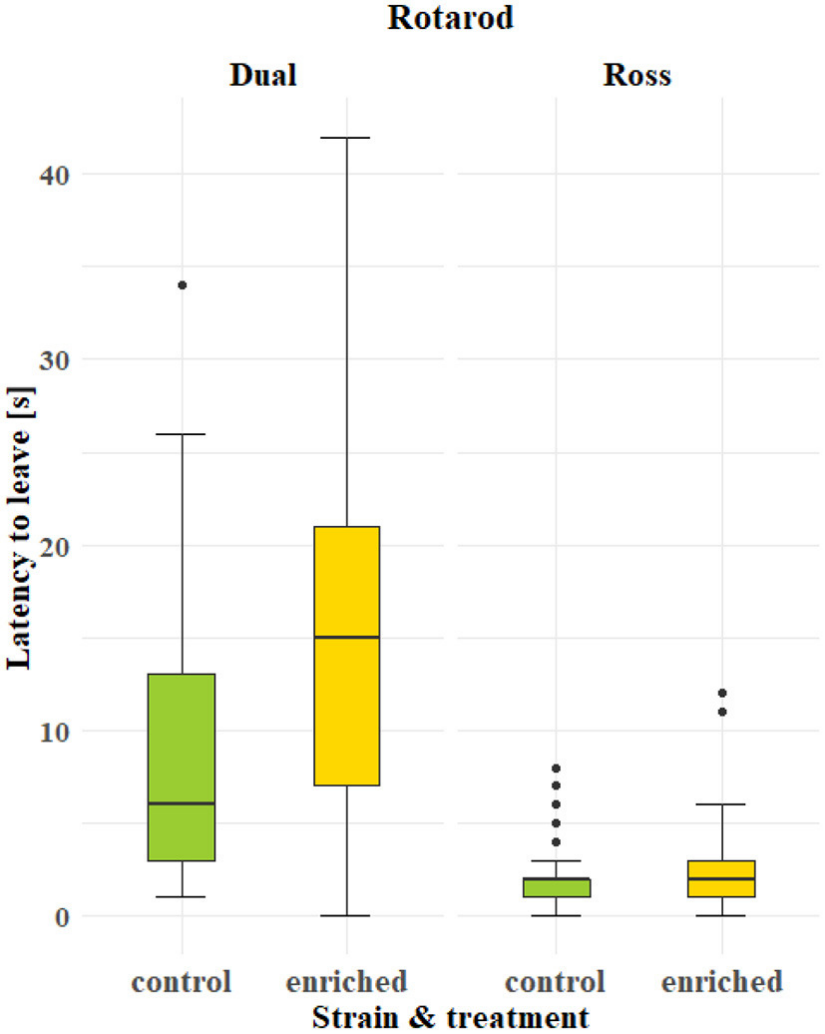
Effect of the strain (Dual or Ross) and treatment (control or enriched) on the latency to leave the rotating rod (boxplots) in the rotarod test.

The latency to leave the rod in the rotarod test was significantly affected by the strain [*F*_(1, 8)_ = 100.4777; *P* < 0.0001] and treatment [*F*_(1, 8)_ = 6.5439, *P* = 0.0337, Figure 5; Supplementary Table 3]. Dual chickens showed a better walking ability than Ross chickens, as indicated by longer latencies. Furthermore, Dual chickens from the enriched treatment showed longer latencies to leave the rod compared to Dual chickens from the control group.

Based on the second method used to assess walking ability, which was the gait score system, Dual chickens also showed a better walking ability than Ross chickens (see Table 1). However, based on this method, the walking ability was not affected by the treatment in either of the strains (Ross, *P* = 0.373; Dual, *P* = 0.557; Supplementary Tables 4, 5).

**Table 1:** Proportion of chickens of the two strains (Dual and Ross) showing the respective gait score at the 33rd/34th (Ross) and at the 70th day of life (Dual) (G = gait score, *n* = 114 per strain).

		**G0**	**G1**	**G2**	**G3**	**G4**	**G5**
Dual	Control	57.9%	36.8%	5.3%	0%	0%	0%
	Enriched	50.9%	45.6%	0%	1.8%	1.8%	0%
Ross	Control	0%	15.8%	31.6%	49.1%	3.5%	0%
	Enriched	0%	24.1%	38.9%	35.2%	1.9%	0%

### Weight, Plumage Cleanliness, and Foot Health

The weight of the chickens was affected by the strain (*P* = 0.0027; Supplementary Table 6), and Dual chickens showed a higher weight at the end of their fattening period (10th week of life) than Ross chickens (5th week of life; Table 2).

**Table 2:** Average body weight (means ± SD) of Dual and Ross chickens across and within treatments (control and enriched) at the 35th (Ross) and the 70th day of life (Dual).

**Strain**	**Treatment**	**Average weight (g)**	**±SD**
Dual	Total	2,237.8	232.7
	Control	2,211.0	210.3
	Enriched	2,264.6	250.3
Ross	Total	2,051.7	351.3
	Control	2,089.4	326.4
	Enriched	2,013.3	371.0

The cleanliness of the chest was affected by the strain (*P* < 0.0001) and that of the back was affected by the treatment (*P* = 0.0005, Table 3; Supplementary Tables 7, 8). Compared to Dual chickens, Ross chickens were clearly dirtier on the chest. Chickens from the enriched groups were dirtier on the back than chickens from the control groups, and this was most obvious in Dual chickens.

**Table 3:** Proportions of Dual (70th day of life) and Ross (35th day of life) chickens showing the respective scores for cleanliness (S = score, *n* = 234 per strain).

**Strain**	**Treatment**	**Cleanliness of chest (%)**				**Cleanliness of back (%)**		
		**S0**	**S1**	**S2**	**S3**	**S0**	**S1**	**S2**
Dual	Total	0.4	88.9	10.7	0	35	64.1	0.9
	Control	0.9	91.5	7.7	0	53	47	0
	Enriched	0	86.3	13.7	0	17.1	81.2	1.7
Ross	Total	0.4	23.1	62.5	25.4	61.2	37.1	1.7
	Control	0	5.1	61.5	33.3	68.4	31.6	0
	Enriched	0.9	18.3	63.5	17.4	53.9	42.6	3.5

Chickens of the two strains differed in their foot health. The mean scores for foot pad lesions [score 0: 62% (Ross) vs. 76% (Dual); *P* = 0.01077] and hock burns [score 0: 78% (Ross) vs. 96% (Dual); *P* = 0.0012; Supplementary Tables 9, 10] were higher in Ross chickens than in Dual chickens (Table 4). The treatment affected the foot pad health (*P* = 0.0113). Dual chickens from the control groups showed better foot pad health than birds from the enriched group.

**Table 4:** Proportions of Dual (70th day of life) and Ross (35th day of life) chickens showing the respective scores for foot pad health (S = score, *n* = 234 per strain).

**Strain**	**Treatment**	**Foot pad change (%)**					**Hock burn (%)**				
		**S0**	**S1**	**S2**	**S3**	**S4**	**S0**	**S1**	**S2**	**S3**	**S4**
Dual	Total	76.1	20.1	3.85	0	0	95.7	4.27	0	0	0
	Control	87.2	9.4	3.42	0	0	98.3	1.71	0	0	0
	Enriched	65	30.8	4.27	0	0	93.2	6.84	0	0	0
Ross	Total	62.1	37.5	0.43	0	0	78.4	21.6	0	0	0
	Control	59.8	40.2	0	0	0	77.8	22.2	0	0	0
	Enriched	64.3	34.8	0.87	0	0	79.1	20.9	0	0	0

### Litter Quality

**Figure 6:**
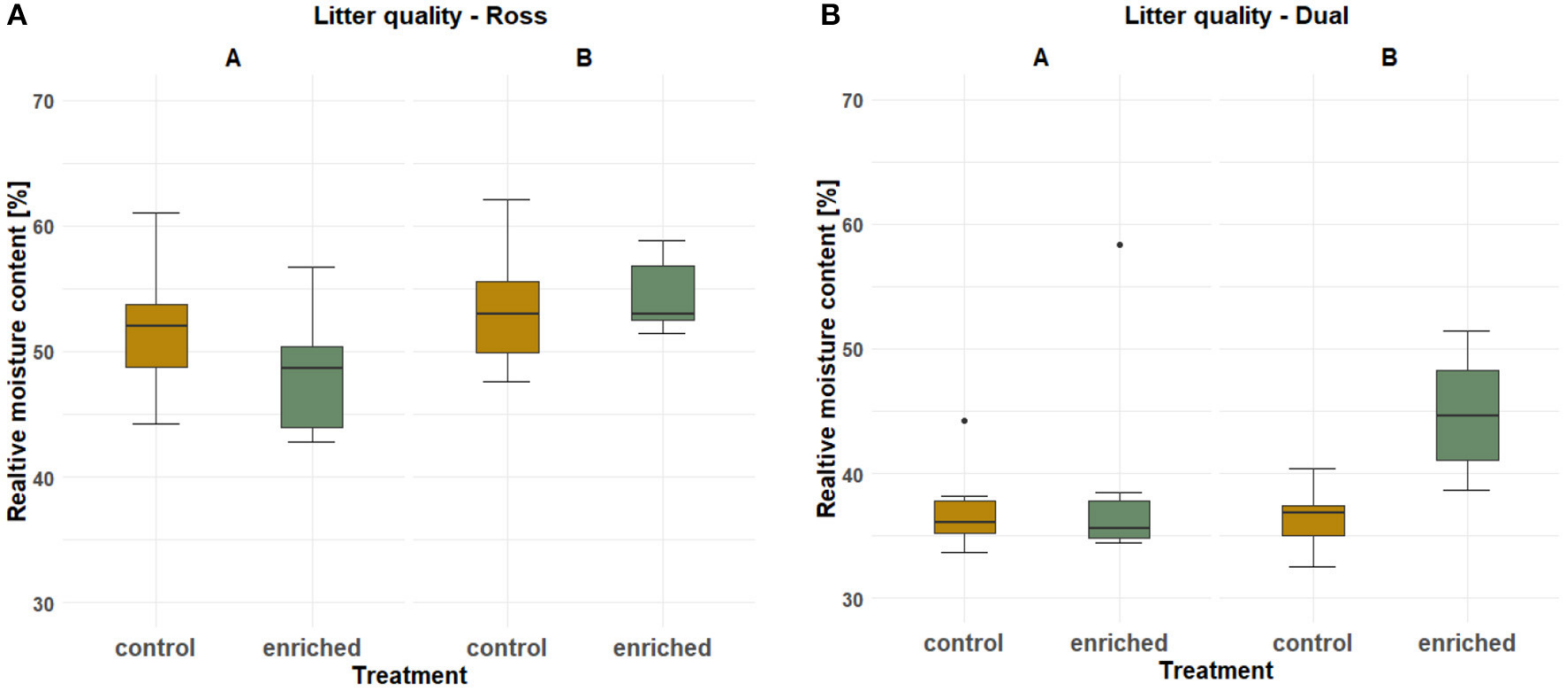
Effect of treatment (control or enriched) and position (A or B, Figure 3) on the litter quality (dry matter content, DM, and boxplots) for each strain [**(A)** Ross and **(B)** Dual], respectively.

The litter quality in the enriched and control compartments was significantly affected by the strain for position “A” (*P* = 0.0002) and by the two-fold interaction between strain and treatment for position “B” (*P* = 0.04). For both strains, the relative moisture content of the litter below the platforms was higher than that of the same area in the control compartments (*P* = 0.013, Figure 6; Supplementary Tables 11, 12).

Within the enriched compartments, litter taken from below the elevated platforms (position “B”) had a higher relative moisture content, i.e., was moister, than the litter taken around the feeding throughs and water dispensers (position “A”) in both strains (*P* = 0.03; Supplementary Table 13). Generally, the litter in the compartments with the fast-growing chickens was moisture than that of the slow-growing chickens.

## Discussion

Our results show that regardless of the growth rate, chickens of both strains used elevated structures offered at a height of 50 cm during both the light and dark periods. However, the frequency of usage differed, and slow-growing chickens used the elevated structures more often than fast-growing chickens. Furthermore, the elevated structures improved the walking ability as measured by the rotarod test in slow-growing but not in fast-growing broilers. Fast-growing broilers had more difficulties in mobility. Their plumage was dirtier on the chest and cleaner on the back compared to slow-growing animals despite their shorter fattening period and lower slaughter weight in this study.

### Usage of Elevated Platforms

The comparison of the two strains showed different intensities of usage of the elevated structures. Beginning in the second week of life, more Dual than Ross chickens were observed on the grids. We expected this difference in usage between the strains, as it has already been described in previous studies (8, 12). With increasing age, chickens of both strains increasingly used the elevated structures. In Ross chickens, this increase was seen until the fourth week, but from the fifth week, usage decreased during the daytime. This decline in usage has also been shown by Norring et al. (21) and is most likely due to rapid growth, a poorly developed bone structure and enormous muscle mass with increasing age (6), which reduces the locomotor activity and ability of these birds (12).

Dual chickens showed higher usage (almost 70%) of the elevated structures from the eighth week of life, particularly at night. Previous calculations (22) showed that the elevated area offered in our study provided space for ~60% of the chickens in a compartment (~32 of 50 animals). In the eighth week of life, the elevated grid area was completely occupied by chickens, and it can be assumed that more space would have led to even more animals on the elevated structures. In pullets, it can also be observed that from the first week of life onwards, most layer chickens look for higher sleeping places (23). There is also a relationship between early access to elevated structures and the frequency of early night-time roosting (24).

Interestingly, male Ross chickens showed higher use of the elevated structures (by up to 17% during the light period and 16% at night) compared to the results of our previous studies (8, 12). In these previous studies, we offered a smaller ramp to reach the grid platforms, and platforms were offered at three different heights (10, 30, and 50 cm). The higher usage in the present study may thus indicate that elevated platforms at a height of 50 cm with a wider, more stable ramp provide better access to the platforms, especially for fast-growing broiler chickens. The area of the elevated grids was narrower than that in the current study but never occupied at full capacity.

In conventional broiler husbandry, male and female broiler chickens are reared in mixed-sex groups. Generally, female chickens are lighter than male chickens (13). There is evidence that lighter chickens use elevated structures more often than heavier chickens (25). Consequently, a higher frequency of usage of elevated structures could be possible in mixed-sex groups. Thus, we suggest that elevated platforms should provide space for ~20% of fast-growing broilers in a barn because it is likely that female birds will use the elevated platform more than male birds. However, this should be validated by on-farm studies in larger groups of broiler chickens, such as Kaukonen et al. (9), to provide more precise details.

### Walking Ability

In both methods for assessing walking ability (rotarod test and gait score system), slow-growing chickens showed a better walking ability than fast-growing chickens. The association between the growth rate and walking ability has also been found in other studies, such as in Kestin et al. (26) and Knowles et al. (27).

An advantage of the rotarod test is that, in this test, the latency to leave the rotating rod as a proxy for walking ability is measured as a continuous variable, in contrast to the gait score system with its categorical variable. In addition, the validity of the gait score system is vulnerable to differences in subjective assessment (15). Thus, with the rotarod test, walking ability can be assessed more sensitively, and smaller differences can be recorded. This is probably the reason why we found an effect of the elevated structures on the walking ability of slow-growing dual-purpose chickens when applying the rotarod test but not with the gait score systems. By using the elevated structures, the motor skills and the walking ability of the chickens of the slow-growing strain were trained, as indicated by a longer latency in the rotarod test. This effect was not prevalent in Ross chickens, as also shown by Bailie et al. (28). In contrast, Kaukonen et al. (9) and Pedersen and Forkman (29) found an improvement in walking ability in fast-growing broiler chickens when elevated structures were offered. The possible training effect of our offered elevated platform does not seem sufficient to improve the walking ability in these fast-growing chickens.

### Plumage Cleanliness and Foot Health

The majority of chickens showed only light to moderate degrees of dirtiness of their plumage. Slow-growing chickens showed cleaner plumage on the chest than Ross broilers. This probably resulted from the lower activity of Ross chickens at the end of the fattening period, when they spend most of their time budget sitting in often moist and soiled bedding (30). In the enriched groups, chickens from both strains showed dirtier back plumage compared to the control groups. The area under the elevated structures was freely accessible to the chickens, and excrement from chickens on top of the elevated structure could fall through the grids. This probably resulted in dirtier backs for the chickens from the enriched compartments compared to the control compartments.

The foot pad health and hocks indicated more lesions in Ross chickens than in slow-growing chickens. This may be due to the lower activity and longer contact time with wet litter in the fast-growing chickens (31). We expected that chickens from enriched compartments showed fewer alterations in the foot pads and hocks. However, the foot pad health and hocks did not differ between the treatments. Either resting on grid platforms affected these measures, or the prevalence of these measures in our study was too low to find any effect of the treatment.

Overall, our results show that natural behaviors such as perching can be supported by offering elevated platforms. In particular, male dual-purpose chickens additionally benefited from the elevated platforms, as indicated by their improved walking ability. The use of such an alternative chicken strain avoids killing day-old male chickens, and in addition, these slower-growing chickens show fewer animal welfare problems than conventional fast-growing broiler chickens. Thus, the use of male chickens of a dual-purpose strain can substantially contribute to improving animal welfare in broiler meat production.

## Data Availability Statement

The original contributions presented in the study are included in the article/Supplementary Material, further inquiries can be directed to the corresponding author/s.

## Ethics Statement

The animal study was reviewed and approved by Lower Saxony State Office for Consumer Protection and Food Safety (LAVES, Oldenburg, Germany) (LAVES, Oldenburg, Germany, file number #33.19-42502-04-16/2108).

## Author Contributions

JM and LS conceived and designed the project. JM collected and analyzed the data. All authors contributed to the manuscript.

## Conflict of Interest

The authors declare that the research was conducted in the absence of any commercial or financial relationships that could be construed as a potential conflict of interest.
