# Supplementary material for: Effects of an Elevated Platform on Welfare Aspects in Male Conventional Broilers and Dual-Purpose Chickens
Source: Front Vet Sci. 2021 May 31;8:660602. doi: 10.3389/fvets.2021.660602 (PMC8200492; doi:10.3389/fvets.2021.660602)
Supplement: Supplementary file 1 [file Data_Sheet_1.PDF]

Supplementary material

Supplementary Table 1 Hatchery and vaccine information

Trial	Strain	Hatchery	Week of laying period	Vaccination (age, name of vaccine)
1	Ross 308	BWE GmbH & Co. KG (Germany)	23	d0: IB Primer<br>d13: ND
	Lohmann Dual	Lohmann Tierzucht GmbH (Germany)	na	d0: Marek HVT & IB Primer<br>d13: ND
2	Ross 308	BWE GmbH & Co. KG (Germany)	17	d0: IB Primer<br>d10: ND
	Lohmann Dual	Lohmann Tierzucht GmbH (Germany)	na	d0: Marek HVT & IB Primer<br>d10: ND

Results

Supplementary Table 2 Usage of the elevated platforms – model output

	numDF	denDF	F-value	p-value
**(Intercept)**	1	163	1577.7985	<0.0001
**Strain**	1	9	199.0895	<0.0001
**LW**	1	163	518.4386	<0.0001
**Time of day**	1	163	6.78	0.0101
**Strain:LW**	1	163	2.8683	0.0923
**Strain:time of day**	1	163	0.8492	0.3581
**LW:time of day**	1	163	26.1246	<0.0001

Walking ability

Supplementary Table 3 Rotarod test – model output

	numDF	denDF	F-value	p-value
**(Intercept)**	1	213	666.2786	<0.0001
**Treatment**	1	8	6.5439	0.0337
**Strain**	1	8	100.4777	<0.0001
**Treatment:strain**	1	8	2.2908	0.1686

Gait Score

Supplementary Table 4 Gait score of Ross – model output

	Chisq	DF	Pr(>Chisq)
**(Intercept)**	105.3537	1	<0.0001
**Treatment**	0.7925	1	0.3733

Supplementary Table 5 Gait Score of Ross – model output

	Chisq	DF	Pr(>Chisq)
**(Intercept)**	10.5353	1	0.001171
**Treatment**	0.3448	1	0.557046

Weight, plumage cleanliness and foot health

Supplementary Table 6 Weight – model output

	numDF	denDF	F-value	p-value
**(Intercept)**	1	442	121914.11	< 0.0001
**Treatment**	1	19	0.13	0.7250
**Strain**	1	19	11.90	0.0027
**Treatment:strain**	1	19	1.27	0.2737

Plumage cleanliness

Supplementary Table 7 Plumage cleanliness of chest – model output

	Chisq	DF	Pr(>Chisq)
**(Intercept)**	0.5468	1	0.4596
**Treatment**	0.2480	1	0.6185
**Strain**	49.0395	1	< 0.0001
**Treatment:strain**	1.8119	1	0.1783

Supplementary Table 8 Plumage cleanliness of back – model output

	Chisq	DF	Pr(>Chisq)
**(Intercept)**	31.339	1	< 0.0001
**Treatment**	12.216	1	0.0005
**Strain**	3.476	1	0.0623
**Treatment:strain**	0.263	1	0.6081

Foot health

Supplementary Table 9 Foot pad – model output

	Chisq	DF	Pr(>Chisq)
**(Intercept)**	22.1521	1	< 0.0001
**Treatment**	6.4213	1	0.01128
**Strain**	6.5037	1	0.01077
**Treatment:strain**	3.5487	1	0.05959

Supplementary Table 10 Hock burn – model output

	Chisq	DF	Pr(>Chisq)
**(Intercept)**	31.0449	1	< 0.0001
**Treatment**	2.5469	1	0.110508
**Strain**	10.4360	1	0.001236
**Treatment:strain**	2.5906	1	0.1075

Litter quality

Supplementary Table 11 Model output of position "A"

	DF	Sum Sq	Mean Sq	F-value	Pr(>F)
**Treatment**	1	0.000099	0.000099	0.0534	0.819666
**Strain**	1	0.040379	0.040379	21.7018	0.0001513
**Treatment:strain**	1	0.002223	0.002223	1.1947	0.2873799
**Residuals**	20	0.037212	0.001861	na	na

Supplementary Table 12 Model output of position "B"

	DF	Sum Sq	Mean Sq	F-value	Pr(>F)
**Treatment**	1	0.013029	0.013029	7.5004	0.01266
**Strain**	1	0.106986	0.106986	61.5875	<0.0001
**Treatment:strain**	1	0.008258	0.008258	4.7540	0.04135
**Residuals**	20	0.034743	0.001737	na	na

S13: enriched "A & B"

Supplementary Table 13 Model output of enriched "A" and "B"

	DF	Sum Sq	Mean Sq	F-value	Pr(>F)
**Treatment**	1	0.024417	0.0244171	14.1636	0.001222
**Strain**	1	0.009355	0.0093554	5.4268	0.030421
**Treatment:strain**	1	0.000006	0.0000061	0.0035	0.953301
**Residuals**	20	0.034479	0.0017239	na	na
